# Self-Assembly and Nanostructures in Organogels Based on a Bolaform Cholesteryl Imide Compound with Conjugated Aromatic Spacer

**DOI:** 10.3390/ma6125893

**Published:** 2013-12-12

**Authors:** Ti-Feng Jiao, Feng-Qing Gao, Xi-Hai Shen, Qing-Rui Zhang, Xian-Fu Zhang, Jing-Xin Zhou, Fa-Ming Gao

**Affiliations:** 1 Hebei Key Laboratory of Applied Chemistry, School of Environmental and Chemical Engineering, Yanshan University, Qinhuangdao 066004, China; E-Mails: gaofengqing619@126.com (F.-Q.G.); shenxihai2007@126.com (X.-H.S.); zhoujingxin@ysu.edu.cn (J.-X.Z.); fmgao@ysu.edu.cn (F.-M.G.); 2 State Key Laboratory of Metastable Materials Science and Technology, Yanshan University, Qinhuangdao 066004, China; 3 College of Physics and Chemistry, Hebei Normal University of Science and Technology, Qinhuangdao 066004, China; E-Mail: zhangxf111@hotmail.com

**Keywords:** nanostructure, self-assembly, organogel, bolaform amphiphile, cholesteryl compound, spacer

## Abstract

The self-assembly of small functional molecules into supramolecular structures is a powerful approach toward the development of new nanoscale materials and devices. As a class of self-assembled materials, low weight molecular organic gelators, organized in special nanoarchitectures through specific non-covalent interactions, has become one of the hot topics in soft matter research due to their scientific values and many potential applications. Here, a bolaform cholesteryl imide compound with conjugated aromatic spacer was designed and synthesized. The gelation behaviors in 23 solvents were investigated as efficient low-molecular-mass organic gelator. The experimental results indicated that the morphologies and assembly modes of as-formed organogels can be regulated by changing the kinds of organic solvents. Scanning electron microscopy and atomic force microscopy observations revealed that the gelator molecule self-assemble into different aggregates, from wrinkle and belt to fiber with the change of solvents. Spectral studies indicated that there existed different H-bond formations between imide groups and assembly modes. Finally, some rational assembly modes in organogels were proposed and discussed. The present work may give some insight to the design and character of new organogelators and soft materials with special structures.

## 1. Introduction

In recent years, Organogels have attracted a great deal of interest due to wide applications on materials, drug delivery, agents, sensors, water purification, *etc*. [1,2,3,4,5,6,7,8,9,10,11,12,13,14,15,16,17,18,19,20,21]. In nature, organogels are various three-dimensional aggregates with micrometer-scale lengths and nanometer-scale diameters immobilizing the flow of liquids. The main driving forces responsible for the formation of supramolecular organogels are specific or non-covalent interactions, such as the dipole-dipole interaction, van der Waals forces, hydrogen bonding, π–π stacking, and host-guest interaction [22,23,24,25,26,27]. Interestingly, complementary hydrogen bonding patterns play a very important role in forming various nanoarchitectures, and their application in the fabrication of organogels has been investigated [28,29,30]. In addition, although gels are originally found in polymer systems, there has recently been an increasing interest in low-molecular mass gelators (LMMGs) [31,32,33]. Such organogels have some advantages over polymer gels: the molecular structure of the gelator is defined, and the gel process is usually reversible. Such properties make it possible to design various functional gel systems and produce more complicated and controllable nanostructures [34,35,36,37].

As an important class of LMMGs, cholesteryl-based imide compounds have been extensively studied for more than two decades, and even today they still remain as an actively investigated class of compounds [38,39,40]. The mentioned “imide structure” is referred to “O=C–NH”, which have demonstrated an important role in forming strong hydrogen bonding and regulating organized aggregates. These organogels have unique directional self-association through van der Waals interactions in the aggregates of the gelators. For example, Shinkai *et al*. prepared a number of dicholesterol derivatives bearing various functional linkers as versatile gelators [41,42,43,44,45] and obtained inorganic materials possessing unique structures by using the corresponding gels as templates. Recently, Fang’s group reported some gel systems, with functionalized cholesteryl derivatives as gelators, and especially, some of the gelators’ gel oil from oil-water mixtures [46,47,48,49]. In addition, in our reported works, the gelation properties of some cholesterol imide derivatives consisting of different substituent groups have been investigated [50,51,52]. We found that a subtle change in the headgroups, spacers, and molecular shapes in molecular skeletons could produce a dramatic change in the gelation behaviors of all compounds.

In this paper, as a continuous work, we have designed and synthesized this bolaform cholesteryl imide compound with large conjugated aromatic spacer. In this compound, the phenanthridine segment with additional phenyl group in spacer was symmetrically attached to cholesterol substituent headgroups to show bolaform molecular skeleton. According to previous reports, 3,8-diamino-6-phenylphenanthridine, as phenyl-phenanthridine dye with a large π-conjugated structure and strong luminescence, were used as materials to synthesize some nanoparticles and show special aggregates [53]. So in present work, this unit was added to the spacer in bolaform amphiphile in order to perform some new characters in organogels in line with those of amphiphiles with hydrophobic or hydrophilic spacers [51]. We have found that the resulting compound was an efficient gelator and could form different organogels in organic solvents. Characterization of these organogels by scanning electron microscopy (SEM) and atomic force microscopy (AFM) revealed different nanostructures of the aggregates in the gels. We have investigated the assembly modes in the microstructures of such organogels and found different kinds of hydrogen bond interactions between imide groups and assembly units.

## 2. Results and Discussion

### 2.1. Gelation Behaviors

**Figure 1:**
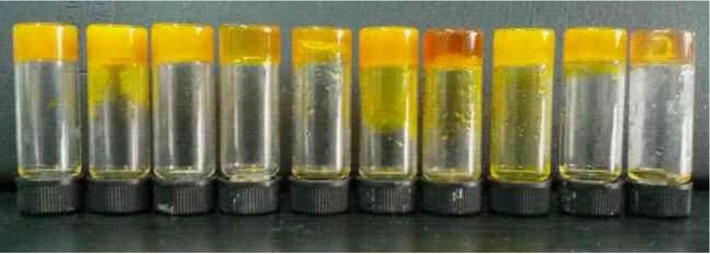
Photographs of organogels of CH-PY in different solvents: n-butanol, n-pentanol, isopentanol, isooctanol, cyclopentanone, cyclohexanone, nitrobenzene, n-butyl acrylate, THF, and DMF (from left to right).

Firstly, the gelation performances of the imide compound abbreviated as CH-PY in 23 solvents are listed in Table 1. Examination of the table reveals that the compound is an efficient gelator. CH-PY can gel in 10 kinds of solvents, such as n-butanol, n-pentanol, isopentanol, isooctanol, cyclopentanone, cyclohexanone, nitrobenzene, n-butyl acrylate, dimethylformamide (THF), and tetrahydrofuran (DMF). The corresponding photographs of organogels of CH-PY in different solvents are shown in Figure 1. The present data shown in Table 1 indicate that the aromatic spacer in molecular skeletons have a profound effect upon the gelation abilities of the present studied imide compound, which is similar to some systems in our previous report about organogels [51]. Therein, it seemed that the suitable combination of flexible/rigid segments in molecular spacer in cholesteryl gelators is favorable for the gelation of organic solvents. In the present case, the intermolecular steric effect and π–π stacking of aromatic spacer for in the gel formation process is also obvious, which may increase the ability of the gelator molecules to self-assemble into ordered structures, a necessity for forming organized three-dimensional network structures.

**Table 1:** Gelation behaviors bolaform cholesteryl compound CH-PY at room temperature.

Solvents	CH-PY	Label
n-Propanol	S	–
Isopropanol	S	–
n-Butanol	G (1.5)	a
n-Pentanol	G (1.5)	b
Isopentanol	G (2.0)	c
Isooctanol	G (2.0)	d
Acetone	PS	–
Cyclopentanone	G (1.5)	e
Cyclohexanone	G (1.5)	f
n-Hexane	PS	–
1,4-Dioxane	PS	–
Benzene	PS	–
Toluene	PG	–
Nitrobenzene	G (2.0)	g
Aniline	S	–
Ethanolamine	I	–
Ethyl acetate	I	–
n-Butyl acrylate	G (1.5)	h
Acetonitrile	I	–
THF	G (2.0)	i
Pyridine	PS	–
Petroleum ether	PS	–
DMF	G (1.5)	j

DMF: dimethylformamide; THF: tetrahydrofuran; S: solution; PS: partially soluble; G: gel; I: insoluble; for gels, the critical gelation concentrations at room temperature are shown in parentheses, (w/v) %.

### 2.2. Morphological Studies of Organogels

**Figure 2:**
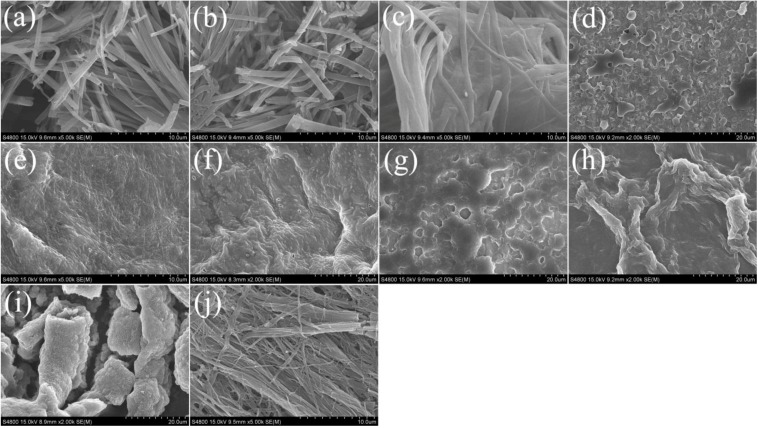
SEM images of xerogels from CH-PY gels: (**a**–**j**) n-butanol, n-pentanol, isopentanol, isooctanol, cyclopentanone, cyclohexanone, nitrobenzene, n-butyl acrylate, THF, DMF, respectively.

It was well-known that a gelator molecule could construct organized nanostructures, such as nanofibers, nanoribbons, and nanosheets in a supramolecular gel [54,55,56,57]. To obtain a visual insight into nanostructures of present gels, the samples of these gels were studied by SEM techniques, as shown in Figure 2. From the present diverse images, it can be easily investigated that the nanostructures of the xerogels in different solvents are significantly different from each other, and the morphologies of the aggregates change from slice, wrinkle, fiber, to rod with change of solvents. Besides, the xerogels of CH-PY in some solvents, such as n-pentanol, cyclopentanone, cyclohexanone, n-butyl acrylate, THF, and DMF, were characterized by AFM, as shown in the supplementary material. From the images, it is interesting to note that morphologies of nanosized fiber, rod, and slice were observed for six xerogels, respectively. At the same time, we have done a comparison test of drying process by freeze-dried method with low temperatures to remove solvents, which can maintain the original structures. The same nanostructures were obtained in both cases. So, the difference of morphologies is attributed to the various assembly modes and forces between gelators and solvents molecules, not the drying process of solvent evaporation. The morphologies of the aggregates shown in the SEM and AFM images may be rationalized by considering a commonly accepted idea that highly directional intermolecular interactions, such as hydrogen bonding or π–π interactions, favor formation of organized belt or fiber micro/nanostructures [57,58,59,60]. The differences of morphologies between different organogels could be mainly due to the different strengths of the hydrophobic force between cholesteryl segments, π–π stacking and steric hindrance between aromatic spacer, which played an important role in regulating the intermolecular orderly staking and formation of special aggregates.

### 2.3. Spectral Studies of Organogels

**Figure 3:**
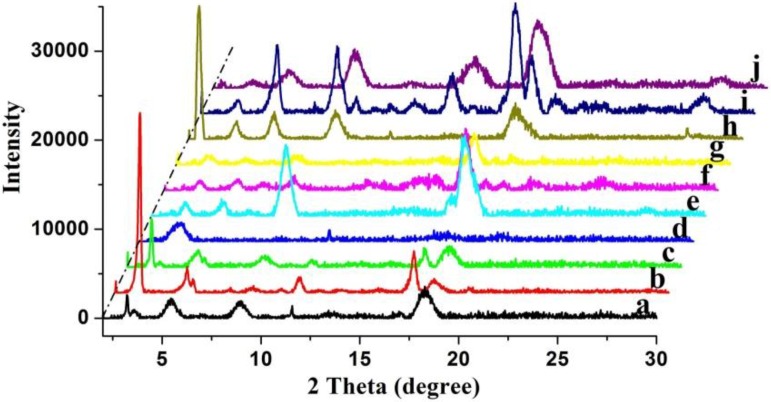
XRD patterns of xerogels from CH-PY gels: (**a**–**j**) n-butanol, n-pentanol, isopentanol, isooctanol, cyclopentanone, cyclohexanone, nitrobenzene, n-butyl acrylate, THF, DMF, respectively.

In addition, with the purpose of investigating the orderly stacking of xerogels nanostructures, XRD of all xerogels from CH-PY gels were measured, as shown in Figure 3. The curve of CH-PY xerogel from n-butyl acrylate showed main peaks in the angle region (20 values, 2.52, 4.40, 6.28, 9.46, and 18.54°) corresponding to *d* values of 3.51, 2.01, 1.41, 0.94, and 0.48 nm, respectively. As for the curves of CH-PY in other solvents, such as n-pentanol and cyclopentanone, the minimum 2θ values are 3.24° and 3.72°, corresponding to *d* values of 2.73 and 2.38 nm, respectively. The change of values can be mainly assigned to the different assembly modes of gelator in various solvents. The XRD results demonstrated again that the solvent had great effects on the assembly modes of the present imide gelator.

**Figure 4:**
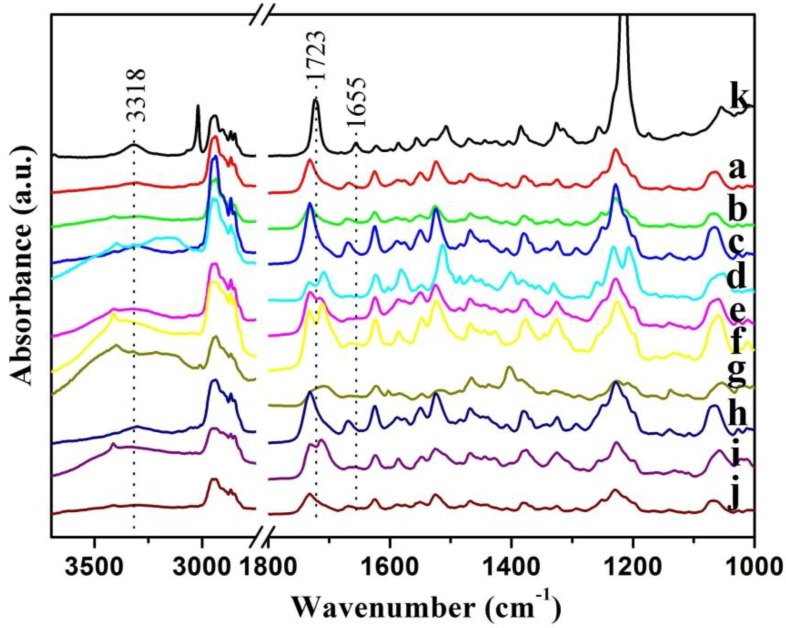
FT-IR spectra of CH-PY in c xerogels (**a**–**j**): n-butanol, n-pentanol, isopentanol, isooctanol, cyclopentanone, cyclohexanone, nitrobenzene, n-butyl acrylate, THF, DMF, respectively, and in chloroform solution (k).

It is well-known that the hydrogen bonding plays an important role in the self-assembly process of organogels [61,62]. At present, we have measured the FT-IR spectra of CH-PY in chloroform solution and xerogels in order to further investigate the assembly process, as shown in Figure 4. As for the spectrum of CH-PY in chloroform solution, four main peaks are observed at 3406, 3318, 1723 and 1655 cm^−1^. These bands can be assigned to the N–H stretching, C=O stretching of ester, and the amide I band, respectively [52,63]. As far as the xerogels from n-butanol, n-pentanol, isopentanol, n-butyl acrylate, and DMF were concerned, these bands shifted to 3301, 1730 and 1664 cm^−1^, respectively. As for the xerogels from other solvents, while these bands shifted to 3294 and 1669 cm^−1^, a new band at 1708 cm^−1^ appeared in these xerogels. The shift of these bands indicates H-bond formation between intermolecular amide and carbonyl groups in the gel state. Spectra of xerogels in different solvents are different, suggesting the different H-bond and assembly modes of gelator in various solvents. This implied that there were differences in the strength and direction of the intermolecular hydrogen-bond interactions in these xerogels. The present data further verified that the organic solvents can regulate the stacking of the gelator molecules to self-assemble into ordered structures by distinct intermolecular hydrogen bonding.

**Figure 5:**
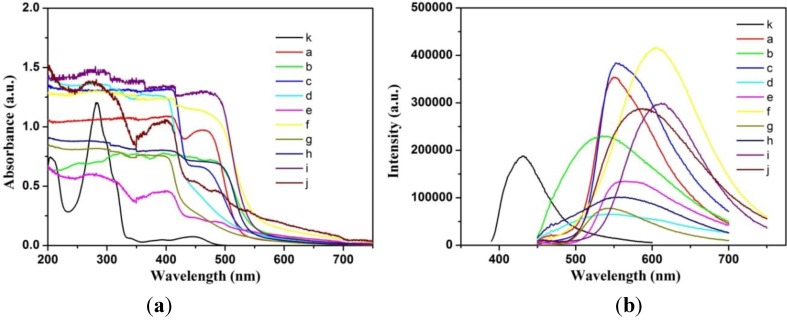
UV-Vis (**a**) and FL (**b**) spectra of CH-PY in xerogels (**a**–**j**): n-butanol, n-pentanol, isopentanol, isooctanol, cyclopentanone, cyclohexanone, nitrobenzene, n-butyl acrylate, THF, DMF, respectively, and in ethanol solution (k).

In addition, the UV-Vis and FL spectra of CH-PY in ethanol solution and xerogels were investigated in order to further character their assembly behaviors, as shown in Figure 5. As for the absorption spectrum of CH-PY in ethanol solution, the absorption peaks appeared at 208, 283, 388, and 447 nm, which could be ascribed to the local π–π* transition of aromatic ring and charge transfer of conjugated structure in spacer [64,65]. For the spectra of xerogels from different solvents, the latter two absorption bands shifted noticeably to the ranges of 398–402 and 465–482 nm, respectively. The spectral changes suggested that CH-PY formed different kinds of J-aggregate-like aggregations in these organized gels. Besides, the FL spectra were also measured to compare this difference. In comparison with the spectrum of ethanol solution with main band at 432 nm, the spectra of xerogels showed main bands in the range of 538–606 nm, indicating different stacking modes of CH-PY in the gels.

### 2.4. Assembly Modes in Organogels

**Figure 6:**
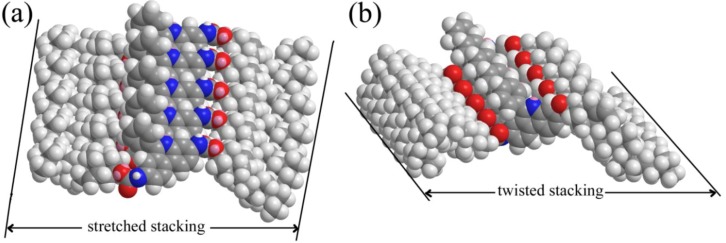
Rational assembly modes of CH-PY organogels in stretched stacking (**a**) 3.5 nm and twisted stacking; (**b)** 2.73 and 2.38 nm, respectively.

Considering the experimental results described above and the hydrogen bonding nature of the organized packing of these organogels as confirmed by FT-IR measurements, some possible assembly modes of the gelator were proposed and schematically shown in Figure 6. As for CH-PY xerogel from n-butyl acrylate, due to the rigidity of conjugated spacer in molecular skeleton and intermolecular forces with solvents, after the intermolecular hydrogen bonding and orderly π–π stacking, CH-PY molecules have a tendency to self-assemble in mode of stretched stacking. So the repeating unit with length of about 3.5 nm was obtained, as show in Figure 6a. As for CH-PY xerogels from other solvents, such as n-pentanol and cyclopentanone, in comparison with π–π stacking, the intermolecular forces with solvents seemed more obvious to adjust molecular conformation to self-assemble and form different twisted stacking nanostructures. The obtained experimental values of CH-PY in n-pentanol and cyclopentanone were 2.73 and 2.38 nm, suggesting the twisted conformation in aggregates, shown in Figure 6b. Meanwhile, it should be noted that this phenomenon can be compared with the results of our recent works [50,51]. Therein, functionalized imide derivatives with the different substituent headgroups or spacers, can have a profound effect upon the gelation abilities and the as-formed nanostructures of the studied compounds. Suitable combination of flexible/rigid segments in molecular spacer in cholesteryl gelators is also favorable for the gelation of organic solvents. For the present gelator, the experimental data showed that the conjugated aromatic spacer in the molecular skeleton played a crucial role in the gelation behavior. The strong π–π stacking and flexible spatial conformation of benzene ring in spacer, as well as in intermolecular forces with solvents, can make the molecules align and stack in an organized way to form various nanostructures. Now, the drug release behaviors generated by the present xerogels in the mixture of Congo red are under investigation to display the relationship between the molecular structures, as-formed nanostructures and properties.

## 3. Experimental Section

### 3.1. Materials

**Figure 7:**
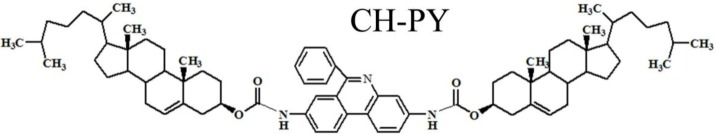
Structure and abbreviation of bolaform cholesteryl imide derivative with aromatic spacer.

The starting materials, cholesteryl chloroformate and 3,8-diamino-6-phenylphenanthridine were purchased from Alfa Aesar (Tianjin, China) and Chemicals and TCI Chemicals (Shanghai, China), respectively. All other used reagents shown in Table 1, sourced from Beijing Chemicals, were used for analysis purity and were distilled before use. Deionized water was used in all cases. Then, the cholesteryl imide compound was synthesized by similar method according to our previous report [40]. Simply speaking, the synthesis of cholesteryl derivative (abbreviated as CH-PY) was carried out from mixing cholesteryl chloroformate with 3,8-diamino-6-phenylphenanthridine in dried dichloromethane with 2.05:1 molar ratio at the presence of pyridine. After the reaction mixture was stirred for 50 h at room temperature, the mixture was washed with dilute hydrochloric acid and pure water, respectively. The organic layer was evaporated to dryness. The product was purified by recrystallization in ethanol solution as yellow solid. The final product and its abbreviation are shown in Figure 7, which were confirmed by ^1^H-NMR and elemental analysis. Ch-PY: ^1^H-NMR (400 MHz, CDCl_3_): δ (ppm) 0.69–1.97 (82H, m, cholesteryl protons), 2.29–2.51 (4H, d, CH_2_), 4.64 (2H, m, oxycyclohexyl), 5.40 (2H, s, alkenyl), 6.76 (2H, s, CONH), 7.64 (2H, s, benzene ring), 7.83 (2H, s, benzene ring), 8.10 (2H, s, benzene ring), 8.52–8.84 (5H, m, benzene ring). Anal. Calcd. for C_75_H_103_N_3_O_4_: C, 81.11; H, 9.35; N, 3.78. Found: C, 81.45; H, 9.42; N, 3.79.

### 3.2. Gel Preparation

The present cholesteryl imide derivative with aromatic spacer was tested to prepare possible organogels according to a simple procedure. Firstly, a weighted amount of imide compound and a measured volume of selected pure organic solvent were placed into a sealed glass bottle and the solution ultrasonicated in a sonic bath for 15 min in order to obtain good dispersion. After that, the solution heated in a water bath at temperatures of 80 °C for 15 min. Then, the solution was cooled to room temperature in air and the test bottle was inversed to see if a gel was formed. At this stage, G, S, PS, and I were denoted to character the states of imide derivative, indicating gel, solution, a few precipitate and insoluble systems, respectively. Critical gelation concentration refers to the minimum concentration of the gelator for gel formation.

### 3.3. Characterization

These prepared organogels under the critical gelation concentration were dried by a vacuum pump for more than 12 h to remove solvents and form xerogels. Then, the obtained xerogel samples were attached to different substrates, such as mica, copper foil, glass, and CaF_2_ slice for morphological and spectral investigation, respectively. AFM data were measured by using Nanoscope VIII Multimode Scanning Probe Microscope (Veeco Instrument, Plainview, NY, USA) with silicon cantilever probes. All AFM images were shown in the height mode without any image processing except flattening. SEM images of the xerogels were measured on a Hitachi S-4800 field emission scanning electron microscopy with the accelerating voltage of 5–15 kV. For SEM measurement, the samples were coated on copper foil fixed by conductive adhesive tape and shielded by gold nanoparticles. The XRD was measured by using a Rigaku D/max 2550PC diffractometer (Rigaku Inc., Tokyo, Japan) with CuKα radiation wavelength of 0.1542 nm under a voltage of 40 kV and a current of 200 mA. UV–vis spectra were obtained by a Jasco UV-530 spectrophotometer (Jasco Corp., Tokyo, Japan). Fluorescence spectra were acquired on a FLS 920 spectrofluorometer (Edinburgh Instruments, Edinburgh, UK) using 1 cm quartz cuvettes or quartz plates. All fluorescence spectra were corrected for the sensitivity of the photo-multiplier tube. FT-IR spectra were obtained by Nicolet is/10 FT-IR spectrophotometer from Thermo Fisher Scientific Inc. by an average 32 scans and at a resolution of 4 cm^−1^.

## 4. Conclusions

In summary, a bolaform cholesteryl imide compound with conjugated aromatic spacer has been designed and synthesized. The gelation behaviors in 23 kinds of organic solvents have been investigated. The morphologies and assembly modes of formed organogels can be regulated by changing organic solvents. Morphological studies revealed that the gelator molecules self-assemble into different aggregates, from wrinkle and belt to fiber with the change of spacers and solvents. Spectral studies indicated that there existed different H-bond formations between imide groups and assembly modes, depending on the solvents. The prepared nanostructures have potential applications in nanomaterial fields. These results afford useful information for the design and development of new versatile low molecular mass organogelators and soft matter.

## Supplementary Materials

Supplementary File 1
