# Supplementary material for: Self-Assembly and Nanostructures in Organogels Based on a Bolaform Cholesteryl Imide Compound with Conjugated Aromatic Spacer
Source: Materials (Basel). 2013 Dec 12;6(12):5893–906. doi: 10.3390/ma6125893 (PMC5452747; doi:10.3390/ma6125893)

# Supplementary Information

**Figure S1.** AFM images of xerogels from CH-PY gels: (**a**–**f**) n-pentanol, cyclopentanone, cyclohexanone, n-butyl acrylate, THF, DMF, respectively. The scan bar is 10 μm.

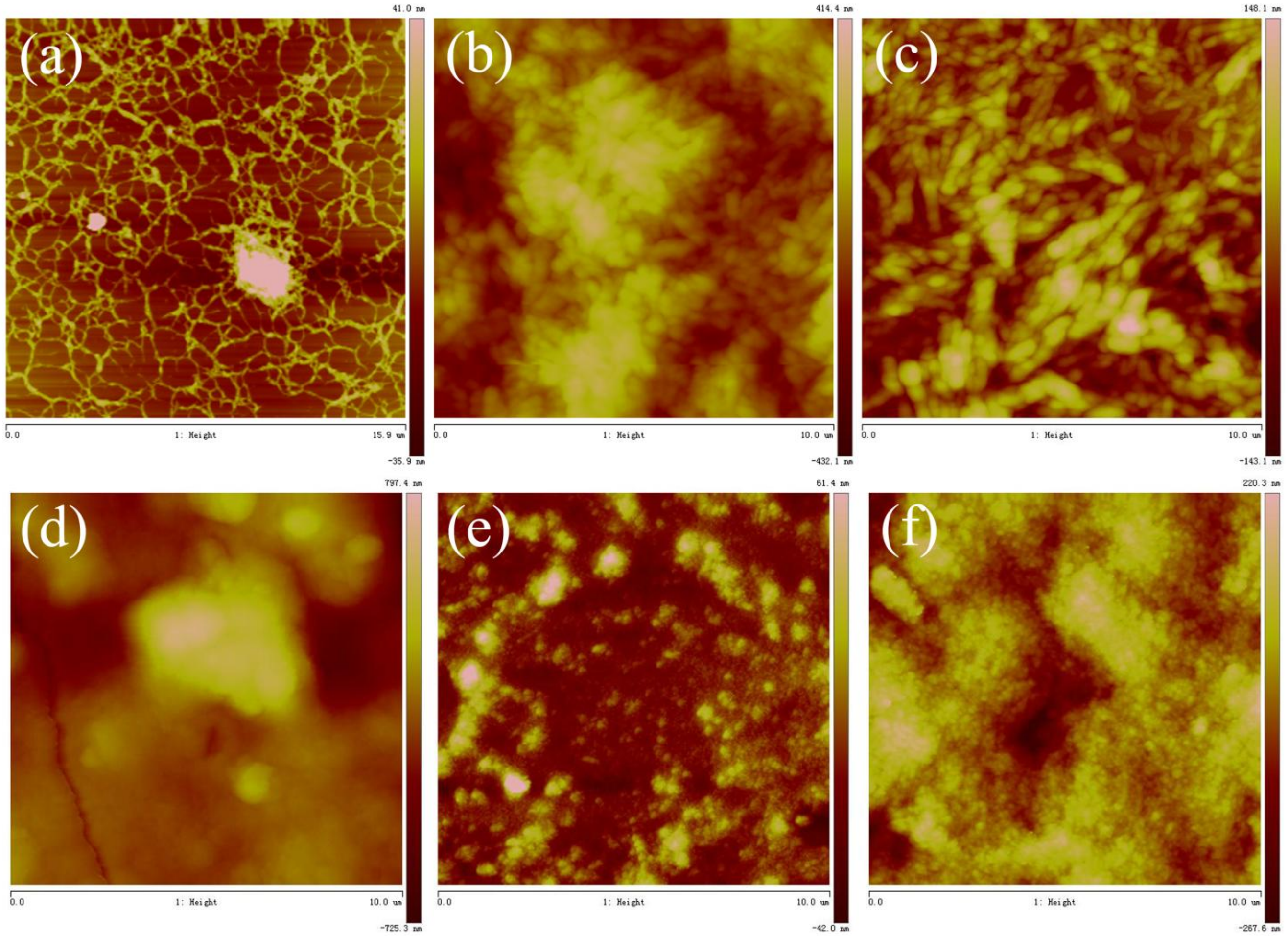

Supplement: Supplementary File 1 [file materials-06-05893-s001.pdf]
